# Supplementary material for: The causal role of circulating inflammatory markers in osteoporosis: a bidirectional Mendelian randomized study
Source: Front Immunol. 2024 Jul 18;15:1412298. doi: 10.3389/fimmu.2024.1412298 (PMC11291241; doi:10.3389/fimmu.2024.1412298)
Supplement: Supplementary file 1 [file DataSheet_1.zip › supplementary material/CIM-OSTEOPOROSIS /GCST90274801.csv_leave_one_out.pdf]

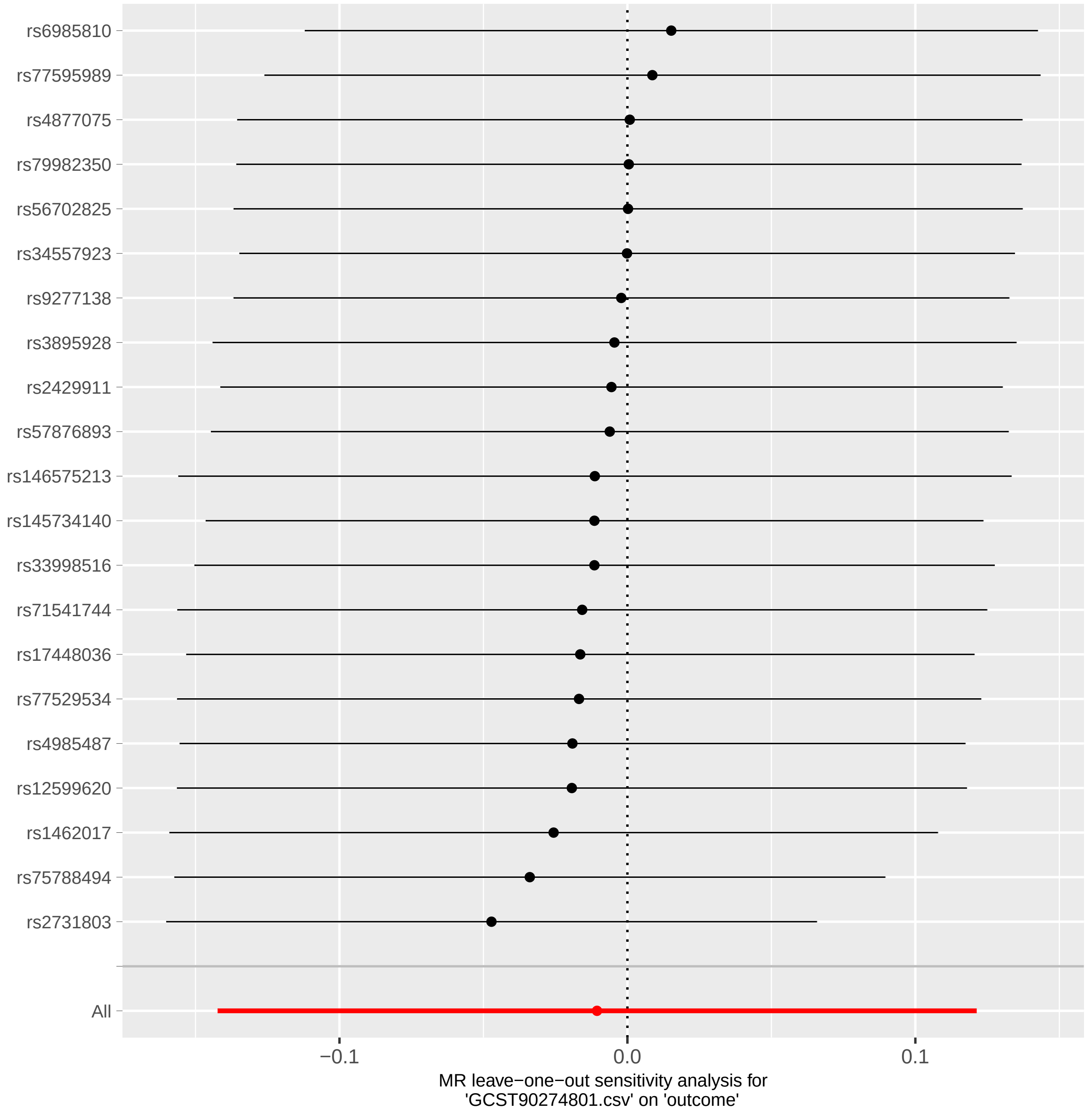
rs6985810
rs77595989
rs4877075
rs79982350
rs56702825
rs34557923
rs9277138
rs3895928
rs2429911
rs57876893
rs146575213
rs145734140
rs33998516
rs71541744
rs17448036
rs77529534
rs4985487
rs12599620
rs1462017
rs75788494
rs2731803
All
−0.1
0.0
0.1
MR leave−one−out sensitivity analysis for
'GCST90274801.csv' on 'outcome'
